# Conditions for laryngeal mask airway placement in terms of oropharyngeal leak pressure: a comparison between blind insertion and laryngoscope-guided insertion

**DOI:** 10.1186/s12871-018-0674-6

**Published:** 2019-01-05

**Authors:** Go Wun Kim, Jong Yeop Kim, Soo Jin Kim, Yeo Rae Moon, Eun Jeong Park, Sung Yong Park

**Affiliations:** 1 Department of Anaesthesiology and Pain Medicine, Ajou University School of Medicine, 164, World Cup-ro, Yeongtong-gu, Suwon, 16499 Republic of Korea; 2 Office of Biostatistics, Ajou University School of Medicine, Suwon, Republic of Korea

**Keywords:** Laryngeal masks, Blind insertion, Laryngoscopy, Equipment and supplies

## Abstract

**Background:**

Insertion under laryngoscopic guidance has been used to achieve ideal positioning of the laryngeal mask airway (LMA). However, to date, the efficacy of this technique has been evaluated only using fiberoptic evaluation, and the results have been conflicting. Other reliable tests to evaluate the efficacy of this technique have not been established. Recently, it has been suggested that the accuracy of LMA placement can be determined by clinical signs such as oropharyngeal leak pressure (OPLP). The aim of this study was to assess the efficacy of LMA insertion under laryngoscopic guidance using OPLP as an indicator.

**Methods:**

After approved by the institutional ethics committee, a prospective comparison of 100 patients divided into 2 groups (50 with blind technique and 50 with the laryngoscope technique) were evaluated. An LMA (LarySeal™, Flexicare medical Ltd., UK) was inserted using the blind approach in the blind insertion group and using laryngoscopy in the laryngoscope-guided insertion group. The OPLP, fiberoptic position score, whether the first attempt at LMA insertion was successful, time taken for insertion, ease of LMA insertion, and adverse airway events were recorded.

**Results:**

Data were presented as mean ± standard deviation. The OPLP was higher in the laryngoscope-guided insertion group than in the blind insertion group (21.4 ± 8.6 cmH_2_O vs. 18.1 ± 6.1 cmH_2_O, *p* = 0.031). The fiberoptic position score, rate of success in the first attempt, ease of insertion, and pharyngolaryngeal adverse events were similar between both groups. The time taken for insertion of the LMA was significantly longer in the laryngoscope-guided insertion group, compared to blind insertion group (35.9 ± 9.5 s vs. 28.7 ± 9.5 s, *p* < 0.0001).

**Conclusion:**

Laryngoscope-guided insertion of LMA improves the airway seal pressure compared to blind insertion. Our result suggests that it may be a useful technique for LMA insertion.

**Trial registration:**

cris.nih.go.kr, identifier: KCT0001945 (2016-06-17).

## Background

The laryngeal mask airway (LMA) has been used routinely during general anaesthesia replacing endotracheal intubation, or has served as a bridge between endotracheal intubation and the facemask in emergent airway management [1, 2]. The blind insertion technique described by Brain is most widely used [3], but insertion of the LMA is not always smooth and anaesthetic gas leakage and gastric insufflation may occur [1, 4]. For achieving the ideal anatomical position of the LMA, various techniques, including insertion with the use of a laryngoscope, have been described [5, 6]. This technique was designed to control the tongue and displace the epiglottis superiorly so that the LMA can be placed over the tongue at a position below the epiglottis, with minimal resistance from the oral soft tissues [5]. However, except fiberoptic assessment which was based on the anatomic position of epiglottis and vocal cords [5, 6], reliable tests for efficacy of this technique have not been established.

In addition, to assess the airway seal and adequate ventilation of LMA, the value of the fiberoptic scoring system has been questioned [4, 7]. Alternative assessment modalities are needed. Recently, it has been suggested that the accuracy of LMA placement can be determined from clinical signs such as oropharyngeal leak pressure (OPLP). OPLP is commonly measured during LMA insertion to evaluate the degree of airway protection. High OPLPs are desirable as they indicate the feasibility of positive pressure ventilation and the likelihood of successful supraglottic airway placement [1, 7–12]. However, so far, no study has evaluated the efficacy of laryngoscope-guided LMA insertion techniques using OPLP as an indicator.

The aim of this randomised prospective study was to assess and compare the efficacy of blind LMA insertion with that of laryngoscope-guided LMA insertion. We considered that OPLP indicates clinical performance or function of the LMA better than fiberoptic score system does. The primary outcome of this study was the OPLP. The secondary outcomes were the fiberoptic position score, rate of success of first attempt at insertion, time taken for insertion of the LMA, ease of insertion, and the occurrence of any pharyngeal adverse event.

## Methods

This prospective, randomised controlled trial was performed at Ajou University Hospital, Suwon, Republic of Korea, and was approved by the Institutional Ethics Committee (AJIRB-MED-DEO-16-072). After obtaining written informed consent for participation in the study, we enrolled 100 patients (American Society of Anesthesiologists physical status I or II, Age 19–70 years) scheduled to receive general anaesthesia with LMA insertion for elective minor surgery or ambulatory surgery. The exclusion criteria were as follows: (1) a recent history of upper respiratory tract infection, and (2) contraindication to the use of the LMA, such as a body mass index (BMI) ≥ 40 kg/m^2^, symptomatic hiatal hernia, or severe oesophageal reflux disease. The trial is registered in a public trial register (Clinical Research Information Service, CRIS) under the identification number KCT0001945.

The patients were randomly divided into two groups with 50 patients in each, using a random group generator: the blind insertion group (Group 1) and the laryngoscope-guided insertion group (Group 2). Preoperative assessment included Mallampati airway classification. After the patient’s arrival in the operating room, routine monitoring was applied, including electrocardiography, pulse oximetry, and noninvasive blood pressure measurement. Bispectral index (BIS) was monitored using a commercial device (A-2000™, Aspect medical systems, USA). Without premedication, anaesthesia was induced using a standard anaesthetic protocol without the use of muscle relaxant. Induction of anaesthesia was achieved with intravenous propofol (1.5–2.0 mg/kg) and remifentanil continuous infusion. Remifentanil infusion was started and maintained at effect-site concentration 2.0 ng/ml. For effect-site target-controlled infusion (TCI) of remifentanil, a commercial TCI pump (Orchestra Base Primea, Fresinus Vial, France) was used. After the patient lost consciousness, with continuous infusion of remifentanil, 2 vol% sevoflurane was administered and mask ventilation was performed for approximately 5 min for adequate depth of anaesthesia and muscle relaxation [13]. When an appropriate depth of anaesthesia was achieved (relaxation of the jaw, BIS < 60), the head was placed in the dorsiflexion sniffing position and a lubricated LMA (LarySeal™, Flexicare medical Ltd., UK) was inserted using the blind technique in Group 1 and under laryngoscopic guidance in Group 2. Selection of the LMA size was based on the body weight of the patient, usually size 3 for women and size 4 for men. Laryngoscopy was performed as described by Campbell et al. [5]; a MacIntosh #3 laryngoscope blade was placed in the vallecula and the epiglottis was identified; then, both the tongue and epiglottis were lifted anteriorly. It was not necessary to visualize the tracheal opening or vocal cords. To ensure optimal inflation of LMA cuff, the LMA cuff was inflated with air and the pressure was stabilized at 60 cmH_2_O using a handheld manometer. Anaesthesiologists who had experience of at least more than 100 insertions of each technique performed the LMA insertion.

The time taken for LMA insertion, ease of LMA insertion, whether the first attempt was successful, OPLP, and fiberoptic position score were recorded. The time taken for LMA insertion was defined as the duration from the time the anaesthesiologist picked up the LMA till the capnography tracing was obtained. A failed attempt was defined as failed passage of the LMA into the pharynx or ineffective ventilation (expiratory tidal volume < 5 mg/kg or absence of a capnography tracing). The second attempt was performed without sniffing position, and if the second attempt failed endotracheal intubation was done. The patients in whom the first insertion was not successful were excluded from the analysis. Following successful LMA placement and ventilation, OPLP was measured by closing the expiratory valve of the circuit at a fixed gas flow rate of 6 L/min and noting the airway pressure at which the gas leaked into the mouth [8]. To ensure safety, the maximal allowable OPLP was fixed at 40 cmH_2_O. Because position of head can impact the OPLP [11], the head and neck were kept in the sniffing position during the study. A fiberoptic scope was passed through the LMA tube to a position 1 cm proximal to the end of the tube, and the fiberoptic position was evaluated using the fiberoptic scoring system [5, 14]: 4, only the vocal cords seen; 3, vocal cords plus posterior epiglottis seen; 2, vocal cords plus anterior epiglottis seen; 1, vocal cords not seen, but function adequate; and 0, functional failure with the vocal cords invisible. The OPLP and fiberoptic position score were documented by an independent researcher, who was blinded to the insertion technique. Upon completion of the study protocol, the anaesthesiologist who performed the LMA insertion provided a subjective assessment of the insertion procedure by grading it as easy, fair, or difficult. Haemodynamic parameters and BIS were recorded at baseline, 1 min after anaesthesia induction, before insertion of the LMA, and 1 min after insertion of the LMA. During the procedures, anaesthesia was maintained with 2 vol% sevoflurane and effect-site TCI of remifentanil at 2.0 ng/ml.

At the end of the surgery, the independent researcher who was blinded to the group allocation removed the LMA after the patient gained consciousness, and collected data on the following adverse events: The presence of blood (none/trace/moderate/severe) after removal of the LMA. The presence or absence of sore throat and dysphonia was assessed at 1 h postoperatively.

### Statistical analysis

The sample size of this study was determined based on previous studies [1, 10]. If the true difference in OPLP between the two groups was 20%, 44 participants would be required in each group to be able to reject the null hypothesis that the population means of the two groups were equal with a probability of 0.8. A total of 100 patients were enrolled considering a 10% dropout rate (α = 0.05, β = 0.8).

We analyzed the data with R software package (R version 3.4.3) and SAS software version 9.4 (2002–2012, SAS Institute Inc., USA). Continuous data were analyzed using Student’s t-test. Nonparametric data were analyzed using the Mann-Whitney test for two independent samples. Nominal data were analyzed using the chi-square test. Haemodynamic data were analyzed using the linear mixed effect model. Data were presented as mean ± standard deviation (SD), or numbers. A *p* value < 0.05 was considered significant.

## Results

**Fig. 1:**
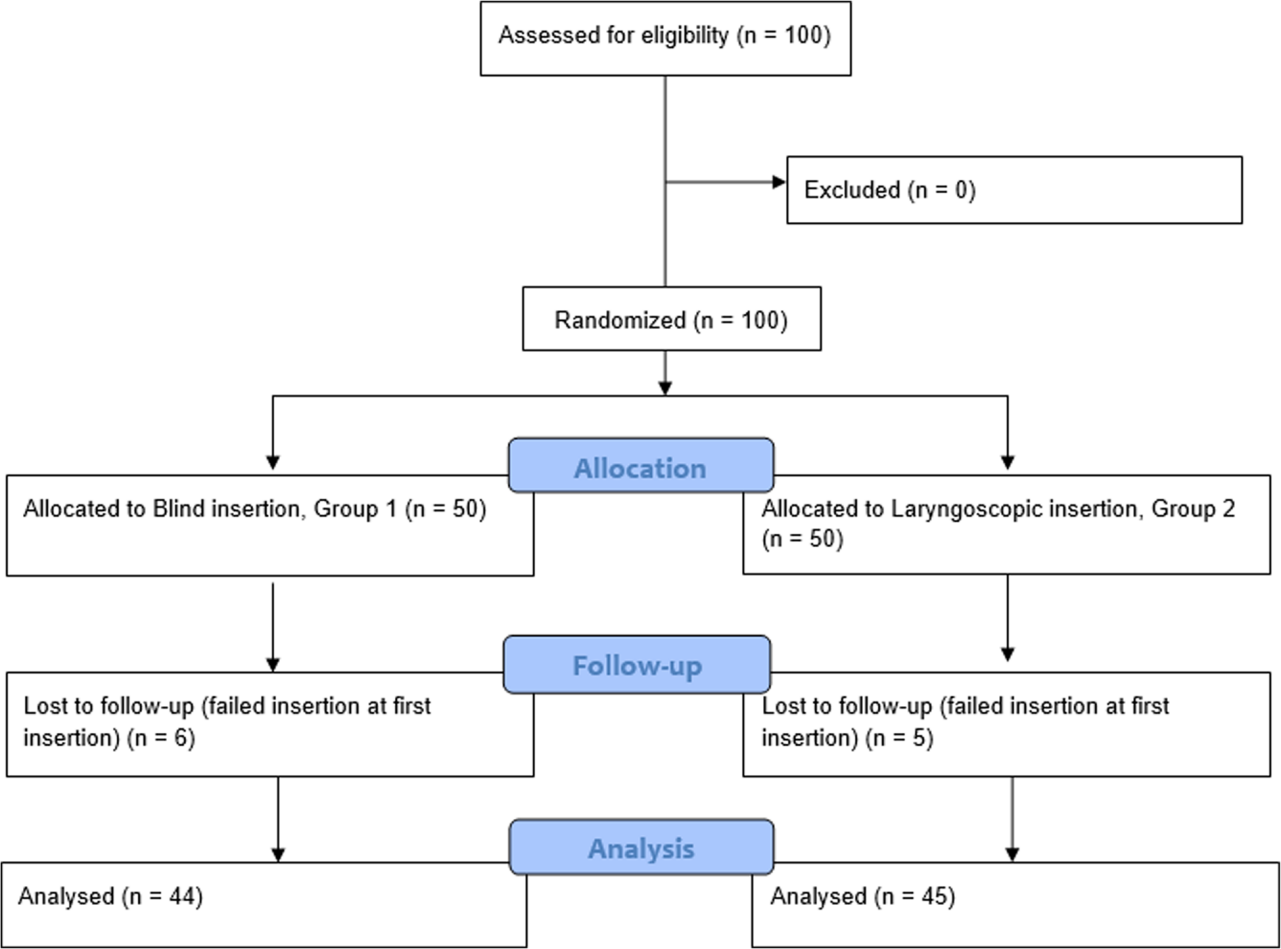
The CONSORT flow diagram

A total of 100 patients consented to participate in the study. The CONSORT flow diagram is shown in Fig. 1. The patient characteristics are shown in Table 1. There were no differences between the two groups in terms of demographic characteristics and Mallampati airway classification. The types of surgery were excision of breast tumor (11), knee arthroscopy (8), removal of fixation device (6), inguinal herniorrhaphy (6), open reduction of fracture (5), debridement (3), others (11) in Group 1, and excision of breast tumor (12), knee arthroscopy (12), removal of fixation device (6), anal surgery (4), ligation of saphneous vein (3), others (13) in Group 2.

**Table 1:** Patient characteristics

	Group 1 (n = 50)	Group 2 (*n* = 50)	*p*
Age (yr)	45.7 ± 12.1	44.9 ± 11.9	.740
Height (cm)	164.4 ± 8.2	164.3 ± 8.8	.981
Weight (kg)	62.7 ± 9.7	63.1 ± 10.7	.847
M/F (n)	24/26	23/27	.841
ASA physical status (I/II, n)	41/9	44/6	.400
Mallampati class (I/II/III/IV, n)	21/20/9/0	23/18/9/0	.907
Surgical time (min)	42.0 ± 23.8	48.3 ± 27.7	.235

Values are presented as mean ± SD or numbers

Group 1 = Blind insertion group; Group 2 = Laryngoscope-guided insertion group; *M* Male; *F* Female; *ASA* American Society of Anesthesiologists

Data on the primary and secondary variables are presented in Table 2. Values were presented as mean ± SD or numbers. The OPLP was higher in the laryngoscope-guided insertion group than in the blind insertion group (21.4 ± 8.6 cmH_2_O vs. 18.1 ± 6.1 cmH_2_O, *p* = 0.031). The fiberoptic position score (*p* = 0.053) and ease of insertion (*p* = 0.405) were not significantly different between the two groups. Rate of success at the first insertion attempt was not significantly different between the two groups (88% in Group 1, 90% in Group 2, *p* = 0.749). In all the patients in whom the first insertion attempt was not successful, success was achieved in the second attempt. The time taken for insertion of the LMA was significantly longer in Group 2 (28.7 ± 9.5 s in Group 1 vs 35.9 ± 9.5 s in Group 2, *p* < 0.0001). There was no difference between groups with respect to adverse events (Table 3). During anaesthesia induction and insertion of the LMA, haemodynamic parameters (mean arterial pressure, heart rate, oxygen saturation) and BIS were not significantly different between the two groups (Table 4). There were no episodes of teeth damage or hypoxia (SaO_2_ < 95%) during the procedures. Anaesthesia was uncomplicated in all patients.

**Table 2:** Safety, efficacy and utility data

	Group 1	Group 2	*p*
Oropharyngeal leak pressure (cmH_2_O)	18.1 ± 6.1	21.4 ± 8.6	.031*
First attempt success rate (n, [%])	44/50 (88%)	45/50 (90%)	.749
Time taken for insertion (s)	28.7 ± 9.5	35.9 ± 9.5	< .0001*
Fiberoptic position score (4/3/2/1/0, n)	15/5/16/7/1	20/10/12/3/0	.053
Easy of insertion (easy/fair/difficult, n)	40/4/6	33/12/5	.405

Values are presented as mean ± SD or numbers

Group 1 = Blind insertion group; Group 2 = Laryngoscope-guided insertion group

* *P* < .05 refers to the statistical difference

**Table 3:** Incidence of adverse events

	Group 1 (*n* = 44)	Group 2 (*n* = 45)	*p*
Bleeding (none/trace/moderate, n)	43/1/0	39/5/1	.053
Pharyngolaryngeal adverse event (none/sore throat/dysphonia)	37/6/1	33/12/0	.189

Values are presented as numbers

Group 1 = Blind insertion group; Group 2 = Laryngoscope-guided insertion group

**Table 4:** Haemodynamic profiles and bispectral indices during laryngeal mask airway insertion

		Baseline	1 min after induction	Pre-insertion	1 min after LMA insertion
MBP (mmHg)	Group 1	96.2 ± 15.0	77.4 ± 12.6	67.2 ± 11.9	71.5 ± 14.0
	Group 2	94.5 ± 12.7	75.4 ± 11.2	65.7 ± 10.8	75.2 ± 12.4
HR (beats/min)	Group 1	72.5 ± 12.7	66.2 ± 11.3	63.5 ± 11.2	63.5 ± 12.3
	Group 2	74.6 ± 11.9	69.2 ± 12.0	65.0 ± 11.5	65.3 ± 12.4
SpO_2_ (%)	Group 1	99.0 ± 1.3	99.9 ± 0.3	99.8 ± 0.6	99.8 ± 0.4
	Group 2	99.0 ± 1.3	99.9 ± 0.4	99.9 ± 0.4	99.8 ± 0.7
BIS	Group 1	96.7 ± 2.4	42.2 ± 10.7	43.2 ± 8.2	48.0 ± 10.9
	Group 2	96.9 ± 1.6	41.3 ± 11.3	44.1 ± 9.9	48.8 ± 11.4

Values are mean ± SD

*LMA* laryngeal mask airway, Group 1 = Blind insertion group; Group 2 = Laryngoscope-guided insertion group; *MAP* mean arterial pressure; *HR* heart rate; *SpO*_*2*_ peripheral oxygen saturation; *BIS* bispectral index

## Discussion

In this study, we demonstrate that laryngoscope-guided insertion of LMA offers an advantage in terms of the OPLP compared with blind insertion. The main finding of our study is that laryngoscope-guided insertion results in a higher OPLP than blind insertion. This suggests that laryngoscope-guided insertion of LMA significantly improves the airway seal pressure. The reason that OPLP was higher in the laryngoscope-guided insertion group is probably because use of direct visual laryngoscopy to facilitate insertion the cuff of LMA plugs more firmly into the periglottic tissue. Direct laryngoscope compresses the tongue to the left so the LMA can be inserted straightforward, minimizing lateral deviation. Under guidance of direct laryngoscope, the LMA can be possibly in alignment with laryngeal skeleton. However, our results indicate that laryngoscope-guided insertion is not superior to blind insertion in terms of achieving proper anatomical placement of the LMA, since the fiberoptic position scores were similar for both techniques.

It is very important to position the LMA correctly in order to ensure proper ventilation and minimize airway adverse events during the LMA insertion. To achieve this, several techniques of LMA insertion have been proposed [5, 6, 15–18], and laryngoscope-guided insertion is one of them [5, 6]. The proposed aim of this technique is to prevent the occlusion of the epiglottis due to insertion of the LMA by lifting the epiglottis upward using the laryngoscope directly during the insertion, so that the epiglottis does not block the vocal cord [5]. To date, the efficacy of this technique has been evaluated using only fiberoptic evaluation, and the results have been conflicting [5, 6]. Campbell et al. [5] used fiberoptic examination to compare the traditional blind insertion technique with direct visual placement using a laryngoscope. They reported that appropriate positioning of the LMA had been achieved in 91.5% of patients in the direct visual placement group, compared with 42% in the blind insertion group; the difference was significant. Chandan et al. [6], however, reported contradictory findings. They reported that there was no statistically significant difference between the blind insertion group and the laryngoscope-guided insertion group using the fiberoptic position scoring proposed by Campbell et al. [5], which is similar to our finding. Because of these conflicting reports, the efficacy of laryngoscope-guided LMA placement, assessed using fiberoptic evaluation, seems to be controversial.

Although the most convenient method to assess the accuracy of anatomic placement of the LMA for clinical studies is fiberoptic examination, the value of the fiberoptic scoring system as a means of assessing proper positioning and airway seal function of the LMA has been debatable [4, 11, 19, 20]. Some studies demonstrated that there was no correlation between position and tightness of the LMA, and no prediction of tightness was possible [7, 20]. In Füllekrug et al.’s study [4], the epiglottis was observed to be in various positions obstructing the glottis opening, but clinical signs of improper placement were rarely observed. The airway can be functionally patent and clinically acceptable even when anatomic placement is less than optimal since the accessory vent allows good airflow to continue. Some researchers have suggested that fiberoptic scoring of the cuff position is not an accurate test to assess the airway seal and ventilation function of the LMA [4, 7, 20]. We speculated that the efficacy of the seal or tightness may vary depending on the individual patient’s laryngopharyngeal anatomy, in addition to the anatomical placement of the LMA.

In contrast to previous studies that evaluated the efficacy of this technique, we measured the OPLP to judge the appropriateness of the airway maintenance and protection, and the correct mounting of the LMA. Recently, it was suggested that the actual tightness of the inserted LMA, rather than fiberoptic view, is an important parameter of adequate airway management [1]. As a mean of this evaluation, an airway sealing pressure or OPLP is commonly performed with the supraglottic airway to quantify the seal with the airway and judge the appropriateness of ventilation [1, 7–12]. OPLP values have been widely used as a reference for feasibility of positive pressure ventilation and the degree of airway protection and is regarded as the most important value when testing the stability of a LMA [9]. Studies to date have not achieved a consensus regarding leak pressure of LMA inserted by laryngoscope-guided technique. To our knowledge, it is the first study that evaluate the usefulness of this technique in terms of OPLP.

Someone might state that the reason to use the blind technique is to avoid the sympathetic stimulation and pharyngolaryngeal adverse events from instrumentation of the oropharyngeal soft tissues during laryngoscopy [5, 6]. Although these haemodynamic changes are short-lived, they are undesirable in patients with pre-existing myocardial or cerebral disease [21]. LMA placement without laryngoscopy avoids airway trauma with fewer changes in haemodynamic parameters [6]. In our study, there were no differences between two groups in haemodynamic parameters and incidence of pharyngolaryngeal adverse events. The reason for these findings was probably secondary to the applied laryngoscopy technique in this study – just gently lift the epiglottis and not necessary to visualize the tracheal opening or vocal cords [5]. It also indicated that the depth of anaesthesia was adequate after 5 min of 2 vol% sevoflurane inhalational induction and remifentanil infusion (effect-site concentration 2.0 ng/ml).

Mean insertion time in laryngoscope-guided insertion group tended to be longer than in blind insertion group (35.9 s vs 28.7 s). Possible reason for longer insertion time in the laryngoscope-guided insertion group was the additional time need for laryngoscopy manipulation procedure. But, we do not consider this to be a clinical problem since it is unlike that the magnitude of this differences have clinical significance.

Our study had some limitations. First, assessment of easy of insertion was not blinded; therefore, this is a possible source of bias. Second, the present study was performed by board-certified anaesthesiologists trained in the use of LMA to minimize the bias due to familiarity with each technique. Therefore, we cannot comment on results obtained by naïve users. Thirdly, our data cannot be applied to the all kinds of supraglottic airway. The type of supraglottic airway (e.g. bulky designed i-gel™ or pre-curved LMA) might also play a very important role if laryngoscopic guidance is better than blind insertion. The variety of cuff’s properties and shapes of supraglottic airway should be considered. Fourthly, we did not use muscle relaxants before insertion of the LMA, because the LMA can be inserted easily without muscle relaxants if an adequate depth of anaesthesia is reached [9, 12]. Because there is some evidence suggesting that the use of neuromuscular blocker can alter the OPLP [22], this should be considered. Finally, the difference between the groups might be statistical different, but 3 cmH_2_O may not make a clinically relevant difference. Some clinicians would argue that OPLP of 18 cmH_2_O is sufficient for most patient in most clinical situations. Although the included number of patients were determined based on previous studies [1, 10], this trial might be underpowered to answer the question of this trial.

## Conclusion

When compared to the blind insertion, laryngoscope-guided insertion of LMA improve the airway seal pressure. Our result suggests that it may be a useful technique for LMA insertion.

## Abbreviations

BIS: Bispectral index
BMI: Body mass index
LMA: Laryngeal mask airway
OPLP: Oropharyngeal leak pressure
TCI: Target-controlled infusion

## References

[1] Seet E, Rajeev S, Firoz T, Yousaf F, Wong J, Wong DT (2010). Safety and efficacy of laryngeal mask airway supreme versus laryngeal mask airway ProSeal: a randomized controlled trial. Eur J Anaesthesiol.

[2] Francksen H, Bein B, Cavus E, Renner J, Scholz J, Steinfath M (2007). Comparison of LMA unique, Ambu laryngeal mask and soft seal laryngeal mask during routine surgical procedures. Eur J Anaesthesiol.

[3] Brain AI (1983). The laryngeal mask-a new concept in airway management. Br J Anaesth.

[4] Füllekrug B, Pothmann W, Werner C, Schulte am Esch J (1993). The laryngeal mask airway: anesthetic gas leakage and fiberoptic control of positioning. J Clin Anesth.

[5] Campbell RL, Biddle C, Assudmi N, Campbell JR, Hotchkiss M (2004). Fiberoptic assessment of laryngeal mask airway placement: blind insertion versus direct visual epiglottoscopy. J Oral Maxillofac Surg.

[6] Chandan SN, Sharma SM, Raveendra US, Rajendra Prasad B (2009). Fiberoptic assessment of laryngeal mask airway placement: a comparison of blind insertion and insertion with the use of a laryngoscope. J Maxillofac Oral Surg.

[7] Brimacombe J, Keller C (2003). Stability of the LMA-ProSeal and standard laryngeal mask airway in different head and neck positions: a randomized crossover study. Eur J Anaesthesiol.

[8] Keller C, Brimacombe JR, Keller K, Morris R (1999). Comparison of four methods for assessing airway sealing pressure with the laryngeal mask airway in adult patients. Br J Anaesth.

[9] Beleña JM, Núñez M, Anta D, Carnero M, Gracia JL, Ayala JL (2013). Comparison of laryngeal mask airway supreme and laryngeal mask airway Proseal with respect to oropharyngeal leak pressure during laparoscopic cholecystectomy: a randomised controlled trial. Eur J Anaesthesiol.

[10] Eschertzhuber S, Brimacombe J, Hohlrieder M, Keller C (2009). The laryngeal mask airway supreme-a single use laryngeal mask airway with an oesophageal vent. A randomised, cross-over study with the laryngeal mask airway ProSeal in paralysed, anaesthetised patients. Anaesthesia.

[11] Kim HJ, Lee K, Bai S, Kim MH, Oh E, Yoo YC (2017). Influence of head and neck position on ventilation using the air-Q® SP airway in anaesthetized paralysed patients: a prospective randomized crossover study. Br J Anaesth.

[12] Gasteiger L, Ofner S, Stögermüller B, Ziegler B, Brimacombe J, Keller C (2016). Randomized crossover study assessing oropharyngeal leak pressure and fiber optic positioning: laryngeal mask airway supreme™ versus laryngeal tube LTS II™ size 2 in non-paralyzed anesthetized children. Anaesthesist.

[13] Joe HB, Kim JY, Kwak HJ, Oh SE, Lee SY, Park SY (2016). Effect of sex differences in remifentanil requirements for the insertion of a laryngeal mask airway during propofol anesthesia: a prospective randomized trial. Medicine.

[14] Brimacombe J (1993). Berry a. a proposed fiber-optic scoring system to standardize the assessment of laryngeal mask airway position. Anesth Analg.

[15] Brimacombe J, Berry A (1993). Insertion of the laryngeal mask airway-a prospective study of four techniques. Anaesth Intensive Care.

[16] Park JH, Lee JS, Nam SB, Ju JW, Kim MS (2016). Standard versus rotation technique for insertion of Supraglottic airway devices: systematic review and meta-analysis. Yonsei Med J.

[17] Jeon YT, Na HS, Park SH, Oh AY, Park HP, Yun MJ (2010). Insertion of the ProSeal laryngeal mask airway is more successful with the 90 degrees rotation technique. Can J Anaesth.

[18] Ghai B, Wig J (2009). Comparison of different techniques of laryngeal mask placement in children. Curr Opin Anaesthesiol.

[19] Joshi S, Sciacca RR, Solanki DR, Young WL, Mathru MM (1998). A prospective evaluation of clinical tests for placement of laryngeal mask airways. Anesthesiology.

[20] Xue FS, Mao P, Liu HP, Yang QY, Li CW, He N (2008). The effects of head flexion on airway seal, quality of ventilation and orogastric tube placement using the ProSeal laryngeal mask airway. Anaesthesia.

[21] Gupta K, Rastogi B, Gupta PK, Singh I, Singh VP, Jain M (2016). Dexmedetomidine infusion as an anesthetic adjuvant to general anesthesia for appropriate surgical field visibility during modified radical mastectomy with i-gel®: a randomized control study. Korean J Anesthesiol.

[22] Goldmann K, Hoch N, Wulf H (2006). Influence of neuromuscular blockade on the airway leak pressure of the ProSeal laryngeal mask airway. Anasthesiol Intensivmed Notfallmed Schmerzther.

